# Assessing hypertension and diabetes knowledge, attitudes and practices among residents in Akatsi South District, Ghana using the KAP questionnaire

**DOI:** 10.3389/fpubh.2023.1056999

**Published:** 2023-06-02

**Authors:** Dorothy O. Asante, Anran Dai, Anita N. Walker, Zhou Zhou, Senam A. Kpogo, Rongzhu Lu, Kaizong Huang, Jianjun Zou

**Affiliations:** ^1^Department of Preventive Medicine and Public Health Laboratory Science, School of Medicine, Jiangsu University, Zhenjiang, China; ^2^School of Basic Medicine and Clinical Pharmacy, China Pharmaceutical University, Nanjing, China; ^3^Department of Clinical Pharmacology, Nanjing First Hospital, Nanjing Medical University, Nanjing, China; ^4^School of Public Health, Nanjing Medical University, Nanjing, China; ^5^School of Nursing and Midwifery, University of Health and Allied Sciences, Ho, Ghana

**Keywords:** hypertension, diabetes, knowledge, attitudes, health education, promotion

## Abstract

**Objective:**

Low awareness of hypertension and diabetes is a public health concern in Ghana. Assessing the general population’s behaviour via knowledge, attitude, and practice (KAP) will be invaluable in these diseases, where prevention and control need a lifelong commitment to a healthy lifestyle. Hence, our goal was to assess the behaviour of Akatsi South residents towards the diseases to assist health providers in implementing tailored intervention programs.

**Methods:**

This was a population-based cross-sectional study with 150 adults (18–70 years) from November to December 2021. A semi-structured questionnaire with face-to-face interviews was used to obtain data. All variables in the model had descriptive statistics. The Chi-square (*χ*^2^) test was used to examine correlations between variables, and a *value of p* < 0.05 was considered statistically significant. The factors associated with checking blood sugar levels and blood pressure were determined using binary logistic regression.

**Results:**

The respondents’ mean age and BMI were 32.40 years (± 12.07) and 24.98 kg/m^2^ (± 2.36), respectively. Only 46.67% of the respondents frequently monitor their blood pressure and 17.33% their blood glucose (at least once a year). Less than half of those surveyed had a good knowledge of hypertension (42.7%) and diabetes (32.0%), whereas nearly 3/4 had poor attitudes regarding both conditions. A binary logistic regression analysis revealed that having a good attitude toward hypertension (exp B = 2.479, *p* = 0.036) and diabetes (exp B = 4.547, *p* = 0.009) were the participants’ strongest predictor of blood pressure and sugar level checks. However, being overweight (exp B = 0.046, *p* = 0.002,) or obese (exp B = 0.144, *p* = 0.034) negatively influenced the frequency with which our respondents checked their blood glucose levels.

**Conclusion:**

In the study, we found that the population generally has poor knowledge, which affects their behaviour (attitudes and practices) towards the diseases. To enable healthcare practitioners to reduce disease-associated mortality and morbidity in the future, frequent public health education and promotion about the conditions is critical to closing the knowledge gap.

## 1. Introduction

Non-communicable diseases (NCDs) are chronic, life-long disorders resulting from a combination of environmental, genetic, behavioural, and physiological factors. Globally, NCDs claim the lives of 41 million people annually, with 77% occurring in low and middle-income countries (LMICs) (1, 2). Hence, they are predicted to overtake infectious diseases as the leading cause of premature mortality and morbidity by 2030 (3, 4). Hypertension (HT) and diabetes (DM) are two of the most common NCDs worldwide and are major risk factors for diseases like stroke and kidney disease. They are considered silent killers because they impair health without showing symptoms (5, 6). According to WHO, an estimated 1.28 billion adults (30–79 years) globally have HT, with 2/3 living in LMICs. The International Diabetes Federation (IDF), on the other hand, revealed that in 2021, 537 million adults (1 in 10 adults aged 20–79) had diabetes, with more than 3/4 coming from LMICs (2, 7).

A significant percentage of the global burden of HT and DM can be attributed to the recent epidemiological shift from communicable to non-communicable diseases across Africa (8), and Ghana is no exception. In Ghana, over one out of four adults (i.e., a prevalence rate of 27%) have HT (9), whereas 2–9% have DM (10, 11), thereby ranking them among the top 10 causes of death in the country (12). Minimizing the number of people who die from early-stage disease is therefore crucial and achievable if individuals have adequate knowledge, good attitudes, and practices towards the illness (13). Studies, however, show that approximately half of the hypertensive and one-third of diabetic individuals have not been diagnosed and are unaware of their conditions, respectively, (14, 15). The prevalence of HT and DM in Ghana varies in urban and rural areas, with low awareness levels. For example, a study in Keta Municipality (which borders our study district) found that rural residents had a higher HT prevalence with lower awareness than urban residents (16).

Specific interventions can be evaluated using knowledge, attitudes, and practices (KAPs) questionnaires. Awareness is the initial disease prevention strategy development stage (5). To assist in implementing initiatives and strategies for successful public health education, exploring the KAPs among the general population is crucial. However, there needs to be more research examining KAPs among the general population, especially in rural areas where HT and DM are on the rise. Those that exist focus on the KAP of diabetic and/or hypertensive patients towards their disease and management, whereas others focus on one condition, particularly hypertension, and its awareness (16). Inadequate or poor knowledge, bad attitudes, and practices toward these diseases will continue to rise as long as the general populace remains uneducated. Hence, exploring KAPs with the general public will help fill the knowledge gap reducing inpatient care and improving clinical outcomes.

As far as we know, research has yet to use KAP questionnaires to investigate the behaviour of the general population regarding HT and DM in a rural Ghanaian community or district. Based on the previous prediction model (17), we explored using simple KAP questions to understand the behaviour of the residents in Akatsi South District towards these diseases to assist healthcare providers in streamlining specific, targeted, and effective intervention programs.

## 2. Materials and methods

### 2.1. Study area

Akatsi South District has a population of 98,684. 46.1% (45,497) of males and 53,187 females (53.9%). However, with a reported 2.4% annual growth rate, the population is predicted to reach 131,174 by 2022 (18). Akatsi is the sole urban town among the 20 main settlements in the district. Rural towns house two-thirds of the district’s population. The district had a crude death rate of 9.2, compared to 8.8 for the region in 2010. There are 29 healthcare facilities with malaria, acute and upper respiratory tract infections, hypertension, rheumatism and joint pains, anaemia, and skin diseases among the top 10 diseases in the district (18, 19).

### 2.2. Study population, exclusion and inclusion criteria

Residents of the twenty major localities (Akatsi, Wute, Agbedrafor, Avenorpedo, Torve, Avenorpeme, Gefia, Dzogadze, Nyitawuta, Dagbamate, Avashive, Tsigbene, Lawui-Avedzi, Klokpe, Apeyime, Xavi, Guiga, Ngblebi, Adeheta, and Atidzive) who were between the ages of 18–70 and gave their consent to participate in the study were included. We excluded those seriously ill, residents who did not consent to participate, and pregnant or nursing mothers from the survey.

### 2.3. Sampling method

A multi-stage sampling strategy is shown in the sections below. We used this strategy to get the data.

#### 2.3.1. Phase 1: choosing a coverage area

The district coverage area was used to determine the study’s baselines and to correlate with prior predictions. The coverage areas were stratified into two groups. Each group included two notable nucleated settlements (Wute, Avenorpeme, Gefia, and Avenorpedo). Group 1 consisted of Akatsi, Wute, Gefia, Klokpe, Avashive, Lawui-Avedzi, Guiga, Ngblebi, Adeheta, and Atidzive, while Xavi, Avenorpedo, Avenorpeme, Torve, Agbedrafor, Dzogadze, Nyitawuta, Dagbamate, Apeyime, and Tsigbene made up Group 2.

#### 2.3.2. Phase 2: choosing a representative sample of people to interview

Households were chosen at random for each area. A point of focus was located within every community, and the interviewer used an iPhone compass to identify their direction while standing at the central location. The interviewer entered the first house facing them. If a household had more than one qualified respondent, one was randomly chosen and interviewed. The exit was followed by a visit to a nearby building whose entrance faced the previous. Interviews were conducted with no more than two eligible individuals (aged 18–70, male or female). We selected Seven first responders from each community. However, in a crowded area like a market, we chose just 2% of the projected eligible population at random. Finally, 150 inhabitants were selected and interviewed based on the same eligibility criteria and sampling method.

### 2.4. Sample size estimation

While a large sample size may raise ethical concerns and time and money waste, a small sample size may cause misrepresentation, inefficiency, and results that are not statistically significant. Because of this, we used the scientific approach to ensure that our sample size was optimum for our study. Ultimately, our sample size met the criteria for efficiency, representativeness, validity, and flexibility (20). To estimate the sample size (21), we used the formula below:


$$n=\frac{z^{2}p\left(q\right)}{d^{2}}$$

Where, z (z score) = 1.96 at 95% confidence level.

d (the precision around the population mean) = 10%.

p (prevalence) = 55% and.

q = (1-p).


$$n=\frac{z^{2}p\left(1-p\right)}{d^{2}}$$

$$n=\frac{1.96^{2}0.55\left(1-0.55\right)}{0.1^{2}}$$

$$n=\frac{1.96^{2}0.55\left(1-0.55\right)}{0.1^{2}}$$

n = 95.

Applying a non-response rate of 5% to the minimum sample size increases the number of participants to 100. As a result, 100 participants were considered the bare minimum for the study. Hence, our sample size of 150 participants was sufficient for the analysis.

### 2.5. Reliability and validity of the questionnaire

#### 2.5.1. Validity of the questionnaire

The District Health Directorate, Ghana Health Service’s NCD prevention and control program, and comparable publications (5, 8, 16) guided the questionnaire’s essential questions. We interviewed three health education and health promotion experts and non-infectious disease experts in a face-to-face and a group discussion to assess the tool’s face validity. Their opinions on the questionnaire’s difficulty, adequacy, the ambiguity of expressions, and insufficiency in meanings were considered.

Four experts in health education, health promotion, and non-infectious diseases were asked for qualitative content validity. After studying the tool, they wrote down their corrections for each item. Content validity was quantified using the content validity ratio (CVR) and index (CVI). The questionnaire’s total CVR score was 0.81–0.84 for knowledge, 0.89 for attitude, and 0.92 for practice, according to 4 experts. The CVI value for all questionnaire sections was derived by averaging the questions of each component, with knowledge 0.84, attitude 0.88, and practice 0.85. The questionnaire’s CVI was 0.86.

#### 2.5.2. Reliability of the questionnaire

To measure reliability, we used a re-test strategy. For this reason, we provided 20 people with the verified questionnaire. After waiting a week, they were given another chance to complete the questionnaires. Overall, the questionnaire’s reliability was 0.86 (0.83 for knowledge, 0.90 for attitude, and 0.86), within the acceptable range. Finally, the tool was a questionnaire with 16 questions divided into three parts.

### 2.6. Data collection

Educated health workers assisted in the data collection. We completed participating member input before data collection—an interviewer-administered a semi-structured questionnaire to collect data for the study. Three sections make up the survey. Section 1: Personal information; Section 2: Anthropometric measurements (weight, height, and BMI); and Section 3: nineteen (19) essential questions: hypertension knowledge (6 questions), hypertension attitude (3), diabetes knowledge (7 questions), diabetes attitude (3).

For the practice aspect, one question each enquiring about the frequency of checking blood pressure and blood glucose was used. The response to the practice was measured with “at least once every year,” “rarely,” or “never checkers.” Then, “at least once every year” were categorized as frequent checkers, while “rarely” or “never checkers” were categorized as infrequent checkers. The knowledge and attitude aspect were collected with a validated and reliable assessment questionnaire based on the **WHO STEPWISE** strategy to monitor non-communicable conditions.

The knowledge covered lifestyle modifications, risk factors, complications, prevention, and/or control. It was evaluated with “True,” “False,” and “I do not know.” Correct responses were coded as 1, while wrong answers were re-coded as 0. The responses were then summed, and a score of 6 for hypertension and 7 for diabetes could be obtained. After this, a score of more than half was denoted as knowledgeable about diabetes or hypertension. It has been observed that the reliability and validity of the knowledge evaluation questionnaire linked to the risk factors for chronic lifestyle illnesses are acceptable. Based on the findings of Frantz (22), the knowledge evaluation questionnaire possesses good and adequate psychometric qualities, as evidenced by its Cronbach Alpha coefficient of 0.80.

The attitude aspect consisted of questions regarding behaviours that could increase or reduce the risk of diabetes and hypertension. This aspect was evaluated with Yes or No. The right attitude towards reducing risk factors of diabetes and hypertension was re-coded as 1, while the opposite was re-coded as 0. The scores were summed, and a participant with a score of at least 2 was regarded as having a good attitude toward the condition. The dependent variables were “checking for blood glucose and blood pressure.” At the same time, the independent variables were knowledge and attitude towards hypertension and diabetes, family history of diabetes, family history of hypertension, and BMI.

#### 2.6.1. Anthropometric measurements

We used a physician’s scale (Zhongshan Camry Electronic Co. Ltd., Guangdong, China) to record the respondents’ weights. Participants were asked to weigh themselves while wearing light clothing and no shoes, and we recorded the results to the nearest 0.5 kilograms (1.1 pounds). To ensure that the data collected was correct, we calibrated all measurement instruments before we used them for the first time.

**Figure 1:**
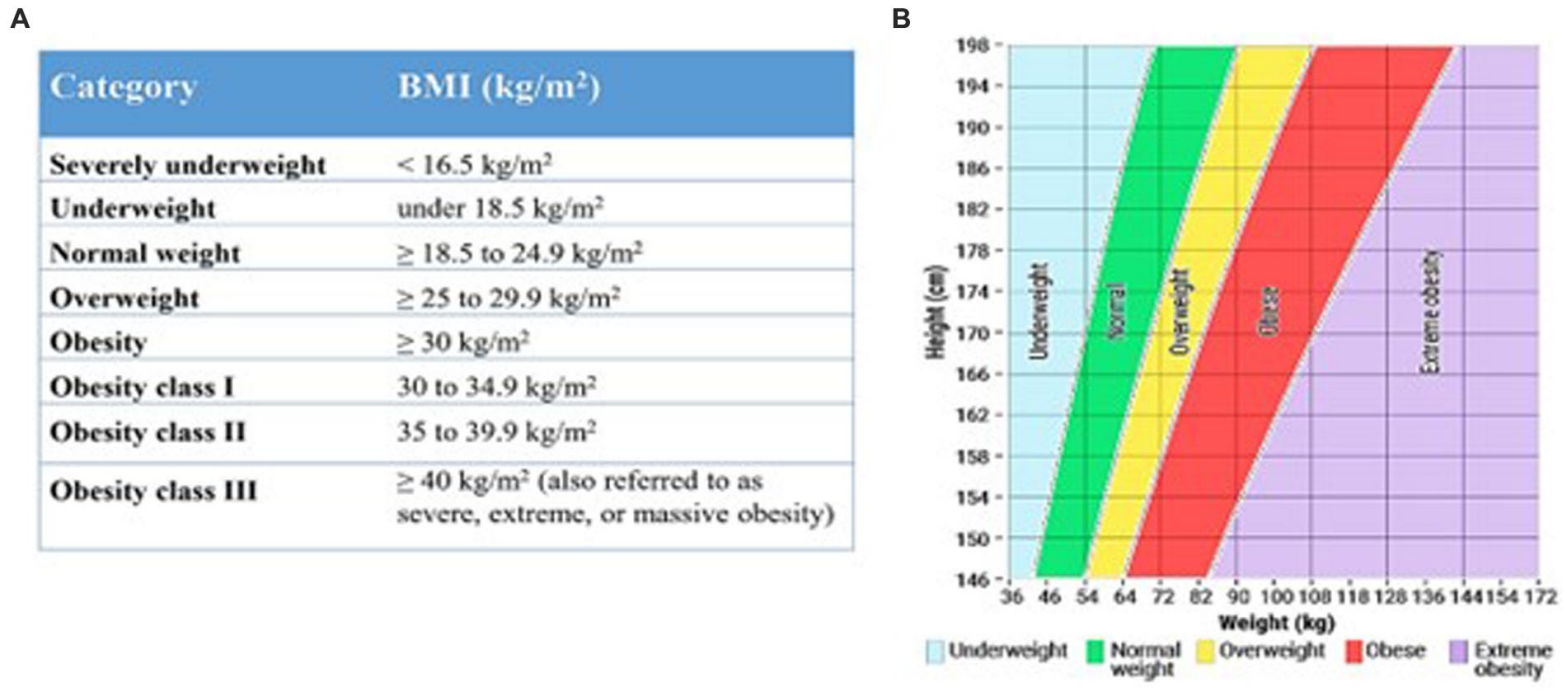
BMI ranges according to the National Institute of Health (NIH) and World Health Organization (WHO) guidelines for white, Hispanic, and black adult individuals.

Participants’ heights were measured accurately to within 0.1 centimeters using a Stadiometer. Weight in kilograms (kg) divided by height in meters squared (m^2^) was used to compute BMI (kg/m^2^). We categorized the BMI ranges according to the National Institute of Health (NIH) and World Health Organization (WHO) guidelines for white, Hispanic, and black adult individuals (23) as shown in Figure 1. Figure 1 shows the (A) Body Mass Index (BMI) table for white, Hispanic, and black people based on National Institute of Health and World Health Organization guidelines, and (B) The Practical Guide: Identification, Evaluation, and Treatment of Overweight and Obesity in Adults (NIH Publication No. 00–4,084) (24).

As the research team was present at all sites, they were obligated to acquire informed permission. The interviewer read and checked the verbal questionnaire with the study respondent. Informed consent was offered in English, the official language, but interpretation in the local language, “Ewe,” was available for clearer understanding. Participants could quit at any time if they felt uncomfortable.

### 2.7. Statistical analysis

Version 26 of the Statistical Package for the Social Sciences (SPSS) was used for data entry and analysis. We provided descriptive statistics for all of the variables in the model. Means and standard deviations were used to summarize quantitative (continuous) data, whereas we used frequencies and percentages for qualitative (categorical) data. The Chi-square (*χ*2) test was used to examine correlations between variables. The factors associated with checking blood sugar levels and blood pressure were determined using binary logistic regression. The main factors established in other literature to affect the checking of blood glucose and sugar level were used in the model; hence, they were not adjusted. A *p* < 0.05 was considered statistically significant.

### 2.8. Ethical considerations and the consent process

Before beginning the study, we requested ethical permission from the Ghana Health Service Ethical Review Committee. Approval number: GHS-ERC: 17/02/2020. The District Health Directorate granted authorization after the researcher informed them of its objectives. We explained the project’s involvement to each participant verbally. There was little or no risk to participants since the study was not intrusive and collected no identifiable information. Participation in this study was free of charge to the participants, and they were not compensated.

## 3. Results

### 3.1. Demographics of the survey participants

Table 1 provides an overview of the demographics of the survey participants. A total of 150 people were interviewed, with the majority of the participants being females, 56.0%. About 54.0% of the participants had basic or primary education while 15.3% had no formal education with less than a quarter (19.3%) having tertiary education. Of most participants, 40% were non-manual workers who engaged in domestic livestock farming, retail sales, crafts, and associated trades, service and sales, and domestic work.

**Table 1:** Demographic characteristics of the respondents in the survey, *N* = 150.

Variable	Frequency (*n*)/Average	Percentage (%)
Gender		
Male	66	44.0
Female	84	56.0
Age	32.40 years	
Level of education		
Not educated	23	15.3
Basic and/primary	81	54.0
Secondary	17	11.3
Tertiary	29	19.3
Occupation		
Students	30	20.0
Unemployed	18	12.0
Manual workers	42	28.0
Non-manual workers	60	40.0
BMI	24.98 kg/m^2^	
Normal (≥18.5–24.9 kg/m^2^)	77	51.3
Overweight (≥25–29.9 kg/m^2^)	66	44.0
Obesity class I (30–34.9 kg/m^2^)	7	4.7
Family history of diabetes		
No	117	78.0
Yes	33	22.0
Family history of hypertension		
No	100	66.7
Yes	50	33.3

Additionally, fewer than one-fourth worked in manual labour, primarily in the fields of skilled agriculture, forestry, and fishing work, as well as carpentry and construction work. But 20.0% were students, mostly at university level, with the majority unemployed or not engaged in any manual or non-manual work. Furthermore, 33.3 and 22.0% had a family history of hypertension and diabetes, respectively. However, 4.7% participants had BMIs in obesity class I, 51.3% had normal BMIs, and over one-third were overweight. Finally, the average age and BMI of the participants were 32.40 years and 24.98 kg/m^2^, with standard deviations of 12.07 and 2.36, respectively.

### 3.2. Respondents’ general knowledge, lifestyle practices and attitudes related to high blood pressure (HBP) and blood glucose level (BGL)

**Figure 2:**
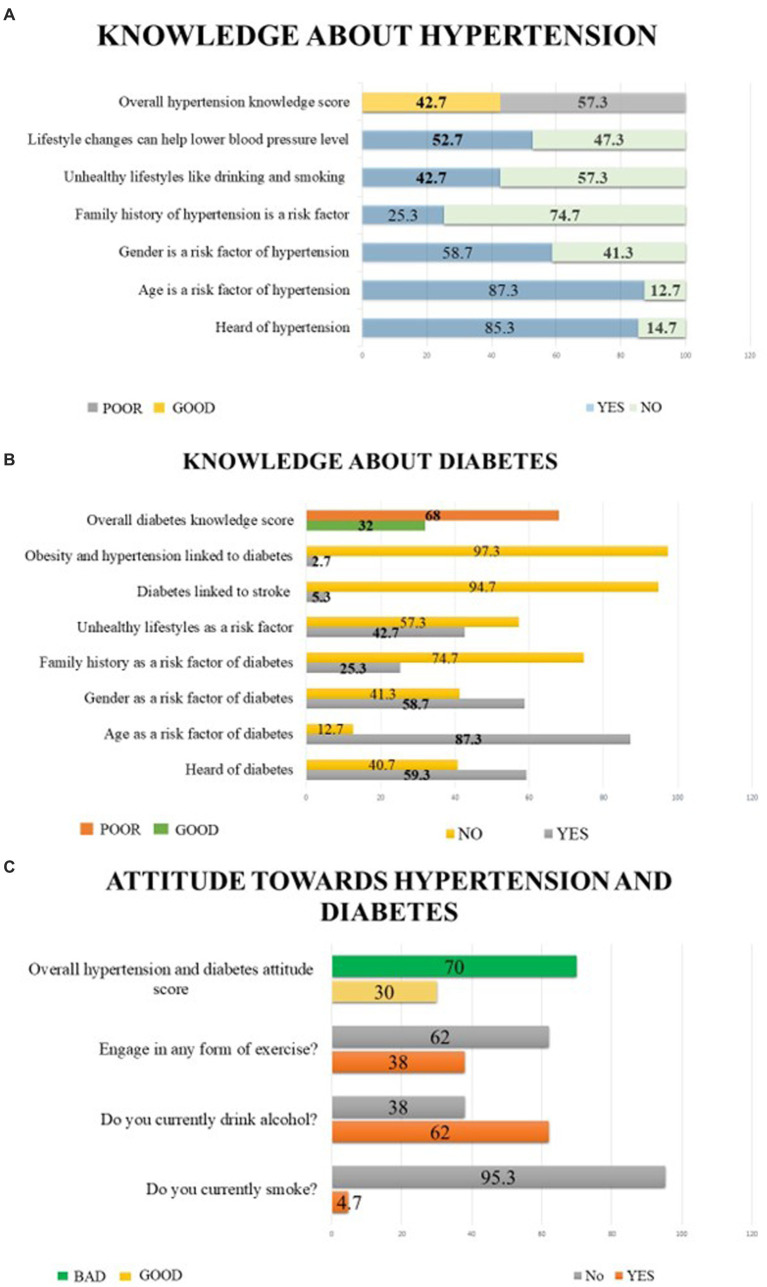
Respondents’ general knowledge, lifestyle practices and attitudes related to high blood pressure (HBP) and blood glucose level (BGL). **(A)** Respondents’ general knowledge and lifestyle practices related to hypertension, **(B)** Respondents’ general knowledge and lifestyle practices related to diabetes, **(C)** Respondents’ general attitude toward both diseases.

Figure 2A shows the respondents’ general knowledge and lifestyle practices related to high blood pressure (HBP), Figure 2B shows the respondents’ general knowledge and lifestyle practices related to blood glucose level (BGL), and Figure 2C show the respondents’ general attitude towards both diseases. 85.3 and 59.3% participants had heard of HT and DM, respectively. Most respondents (87.3%) said that becoming older increases your risk of developing HT and DM. 58.7 and 42.7% of the participants also answered that gender and unhealthy lifestyles are risk factors for both diseases, respectively, with only a quarter of the participants, 25.3%, stating that family history also puts you at risk.

We also indicated lifestyle changes as a measure to help reduce HBP by 52.7% of the respondents. Only 2.7 and 5.3% participants knew obesity and HT are linked to DM, and DM is related to stroke. Furthermore, less than two-fourths of those surveyed were knowledgeable about HT, its modifiable and non-modifiable risk factors, and preventative strategies based on lifestyle changes. On the other hand, just 32% of those surveyed were knowledgeable about DM, its risk factors, and complications. Concerning attitudes towards both diseases, only 4.7% participants smoked, while more than half (62.0%) drank alcohol. Finally, only 38.0% of the surveyed respondents were engaged in some form of exercise. Nearly three-fourths of the participants had bad attitudes regarding HT and DM.

### 3.3. Chi square and Kruskal Wallis test

Table 2 shows the chi-square or Kruskal-Wallis test to see how our respondents’ demographic characteristics, knowledge, and attitudes varied regarding checking for blood pressure and blood sugar levels. Blood pressure checking varied across age (*χ*2 = 8.609, *p* =0 0.003), education (*χ*2 = 5.091, *p* = 0.024), occupation (*χ*2 = 7.484, *p* = 0.009), BMI (*χ*2 = 8.740, *p* = 0.025), family history of diabetes (*χ*2 = 4.592, *p* = 0.032), knowledge about hypertension (*χ*2 = 19.106, *p* < 0.001) and diabetes (*χ*2 = 19.965, *p* < 0.001), and both for attitude towards diabetes and hypertension (*χ*2 = 4.592, *p*-value = 0.032). Here, non-manual workers, those with basic or primary education, those aged 26 years and above, and obese individuals checked their blood pressure frequently. Inversely, more than half of the people who had poor knowledge about diabetes and hypertension, as well as those who had a bad attitude towards hypertension and diabetes, were less likely to monitor their blood pressure.

**Table 2:** Results of how often participants check their blood pressure level (BPL) and blood glucose level (BGL) in a year varies across demographic characteristics, knowledge and attitude using Chi square test or Kruskal Wallis test.

Variable n (%)		Blood pressure checking		*p*-value	Pearson Chi-square/Kruskal Wallis	Blood glucose checking		*p*-value	Pearson Chi-square/Kruskal Wallis
		Rarely/Never n (%) 80 (53.33)	Frequently *n* (%) 70 (46.67)			Rarely/Never n (%) 124 (82.67)	Frequently *n* (%) 26 (17.33)		
*Gender*				0.553	0.352			0.135	2.234
Male	66 (44.00)	37 (56.1)	29 (43.9)			58 (87.88)	8 (12.12)		
Female	84 (56.00)	43 (51.19)	41 (48.81)			66 (78.57)	18 (21.43)		
*Age*				0.003	8.609			*p* < 0.001	23.406
25 years and below	64 (42.70)	43 (67.19)	21 (32.81)			64 (100)	0 (0)		
26 years and above	86 (57.30)	37 (43.02)	49 (56.98)			60 (69.77)	26 (30.23)		
*Educational level*				0.024	5.091			0.025	7.432
Not educated	23 (15.30)	10 (43.48)	13 (56.52)			15 (65.22)	8 (34.78)		
Basic and or primary	81 (54.00)	39 (48.15)	42 (51.85)			67 (82.72)	14 (17.28)		
Secondary	17 (11.30)	11 (64.71)	6 (35.29)			16 (94.12)	1 (5.88)		
Tertiary	29 (19.30)	20 (68.97)	9 (33.03)			26 (89.66)	3 (10.34)		
*Occupation*				0.009	7.484			0.009	11.685
Student	30 (20.00)	22 (73.33)	8 (26.67)			29 (96.67)	1 (3.33)		
Unemployed	18 (12.00)	11 (61.11)	7 (38.89)			18 (100)	0 (0)		
Manual worker	42 (28.00)	20 (47.62)	22 (52.38)			31 (73.81)	11 (26.19)		
Non-manual worker	60 (40.00)	27 (45.00)	33 (55.00)			46 (76.67)	14 (23.33)		
*BMI*				0.025	8.740			0.012	8.790
Normal	77 (51.30)	43 (55.80)	34 (44.2)			67 (87.01)	10 (12.99)		
Overweight	66 (44.00)	35 (53.0)	31 (47.0)			54 (81.82)	12 (18.18)		
Obese	7 (4.70)	2 (28.60)	5 (71.40)			3 (42.86)	4 (57.14)		
*Family history of hypertension*				0.132	2.263			0.446	0.582
Yes	50 (33.30)	31 (62.00)	19 (38.00)			43 (86.00)	7 (14.00)		
No	100 (66.7)	49 (49.00)	51 (51.00)			81 (81.00)	19 (19.00)		
*Family history of Diabetes*				0.032	4.592			0.333	0.444
Yes	33 (22.00)	18 (54.55)	15 (45.45)			26 (78.79)	7 (21.21)		
No	117 (78.0)	62 (52.99)	55 (47.01)			98 (83.76)	19 (16.24)		
*Knowledge about hypertension*				*p* < 0.001	19.106			0.001	2.724
Good	64 (42.70)	30 (46.90)	34 (53.1)			45 (70.3)	19 (29.7)		
Poor	86 (57.30)	50 (58.20)	36 (41.9)			79 (91.90)	7 (8.10)		
*Attitude towards hypertension*				0.032	4.592			0.044	3.908
Bad	105 (70.0)	62 (59.05)	43 (40.95)			91 (86.67)	14 (13.33)		
Good	45 (30.00)	18 (40.00)	27 (60.00)			33 (73.33)	12 (26.67)		
*Knowledge about diabetes*				*p* < 0.001	19.965			*p* < 0.001	4.307
Good	48 (32.00)	20 (41.70)	28 (58.30)			31 (64.60)	17 (35.40)		
Poor	102 (68.0)	60 (58.8)	42 (41.2)			93 (91.20)	9 (8.80)		
*Attitude towards diabetes*				0.032	4.592			0.044	3.908
Bad	105 (70.0)	62 (59.05)	43 (40.95)			91 (86.67)	14 (13.33)		
Good	45 (30.0)	18 (40.00)	27 (60.00)			33 (73.33)	12 (26.67)		

Similarly, except for gender, and family history of hypertension and diabetes, checking of blood sugar levels varied significantly across all other variables. Thus, BMI (*χ*2 = 8.790, *p* = 0.012), attitude towards hypertension and diabetes (*χ*2 = 3.908, *p* = 0.044), age (*χ*2 = 23.406, *p* < 0.001), education (*χ*2 = 7.432, *p*-value =0 0.025), occupation (*χ*2 = 11.685, *p*-value =0 0.009), knowledge about hypertension (*χ*2 = 2.724, *p*-value =0 0.001), and knowledge about diabetes (*χ*2 = 4.307, *p* < 0 0.001). Except for obese individuals, all other individuals were less likely to check their BGL frequently.

### 3.4. Binary logistic regression analysis of factors influencing blood pressure and blood glucose level among participants

In the binary logistic regression analysis in Table 3, having a good attitude toward hypertension (exp B = 2.479, *p* = 0.036) was the strongest predictor of blood pressure checks among our participants. However, being overweight (exp B = 0.040, *p* = 0.010) and being knowledgeable about hypertension (exp B = 0.024, *p* = 0.001) negatively affected checking of blood pressure. On the other hand, as shown in Table 4, BMI group (overweight – exp. B = 0.046, *p* = 0.002) and (obese – exp. B = 0.144, *p* = 0.034) were negatively affected, while having a good attitude toward diabetes positively influenced the frequency with which our respondents checked their blood glucose levels (exp B = 4.547, *p* = 0.009). However, knowing about diabetes or having a family history of the disease had no effect on how often our respondents checked their blood sugar levels.

**Table 3:** Factors influencing checking of blood pressure among participants.

Variables	Sig.	Exp(B)	95% C.I. for EXP(B)	
			Lower	Upper
Gender (females)	0.387	0.730	0.357	1.489
Age (26 years and above)	0.038	2.187	1.043	4.585
BMI group (Normal)	0.036			
BMI group (Overweight)	0.010	0.040	0.015	0.498
BMI group (Obese)	0.061	0.142	0.316	1.199
Knowledge about hypertension	0.001	0.024	0.003	0.167
Have good attitude toward hypertension	0.036	2.479	1.062	5.788
Have family history of hypertension	0.126	0.316	0.271	2.174

**Table 4:** Factors influencing checking of blood glucose level among participants.

Variables	Sig.	Exp(B)	95% C.I. for EXP(B)	
			Lower	Upper
Gender (female)	0.034	0.305	0.102	0.915
BMI group (Normal)	0.008			
BMI group (Overweight)	0.002	0.046	0.006	0.334
BMI group (Obese)	0.034	0.144	0.024	0.860
Knowledge about diabetes	0.065	0.136	0.016	1.131
Have good attitude toward diabetes	0.009	4.547	1.459	14.165
Have family history of diabetes	0.989	1.008	0.335	3.030

## 4. Discussion

Inadequate knowledge, screening, treatment, and prevention of HT, DM, and their complications increase the burden on healthcare systems. Hence, they significantly threaten public health and development (25, 26). Individuals with HT or DM or those at risk require long-term care that is tailored, preventive, and affordable. They are considered silent killers because they do not cause symptoms until the condition is advanced (27). However, despite the rising incidence, many individuals are unaware of their BPL and BGL or associated problems with both diseases. Unless addressed, these health issues’ mortality and morbidity burden will continue to rise.

Validated questionnaires for testing knowledge of hypertension and diabetes were adopted in this study. We noted that general knowledge about both diseases was poor, particularly with diabetes, leading to bad attitudes. Thus, only 32.0 and 42.7% of the surveyed knew about DM and HT, respectively, while nearly three-fourths had bad attitudes towards the diseases, which shows a lot of knowledge gaps concerning the two diseases. The findings of poor knowledge of hypertension and diabetes were consistent with previous studies (5, 28). However, the results of diabetes knowledge were inconsistent with those of Anyanti et al. (8), who reported a high level of knowledge among their research participants. The lack of understanding of the complications of diabetes among our participants could have been attributed to the differences.

Regular BPL and BGL examinations are essential to detect hypertension and diabetes at an early stage. Although gender did not affect BPL and BGL checks, it is to be noted that more females than males checked their BPL and BGL. In addition, similar to the findings of Anyanti et al. from Nigeria (8) the older participants checked their BPL and BGL more frequently than the younger participants. We note a worrisome finding in the BG check, in which no one under 25 checked their BGL regularly. This finding is attributable to the fact that studies have revealed that the risk of cardiovascular diseases, including hypertension and diabetes, rises with age (29). As a result, older people are more likely than younger people to be aware of the importance of regular BP and BG checks.

Additionally, those with high levels of education, and students, were less likely to check their BGL and BPL. However, these results contradict what was found among Nigerians. Similarly, unemployed individuals were also less likely to undertake routine BPL and BGL checks. Lower monthly incomes may hinder people from requesting these tests because of their high cost, and this could be a significant reason why those people would not routinely check their BGL, even though the study did not consider their monthly wage. Furthermore, it is well established that individuals who perceived themselves to be healthy are less likely to undertake medical screening as it was found in our study (30). Nevertheless, the overweight individuals not checking their BGL and BPL is an alarming situation which needs urgent attention as these individuals are more at risk of developing high BP and BG (31).

Furthermore, we discovered that most participants checked their BPL more frequently than their BGL this is similar to what was found in Nigeria (8). Unlike BG checks, BP checks are part of the standard examination for outpatients in Ghana, independent of the patient’s presentation. Nonetheless, less than two-thirds of these people monitor their BP at least once a year, which could be better given the need for regular BP monitoring to prevent hypertension progression.

Ultimately, the findings of this study revealed that neither knowledge of diabetes nor a family history of the disease affected how frequent our respondents checked their blood sugar levels. This contradicts the findings of Anyanti et al. where they found that being knowledgeable and having family history of diabetes made the respondents to check their blood glucose routinely. Our results could also be as a result of the myth in Ghana that, “if I do not know, it will not kill me.” In that, most uneducated Ghanaians believes that, when you are aware of your health condition you begin to think about it which can result in their death., Lastly, we discovered having a positive attitude toward both diabetes and hypertension to be the participants’ primary determinant of blood pressure or sugar checks. Positive attitude toward a condition has been established to improve the measures required to maintain or control the health condition (8, 32). As far as this study is concerned, it did not look at how hypertension and diabetes were related in terms of knowledge, practices, and prevalence because the study design did not include the screening of the participants. While this does not contradict the study’s findings, future research may focus on hypertension and diabetes screening to better establish the relationship between lifestyle and disease prevalence.

### 4.1. Strengths and limitations of the study

This study has several notable strengths. The study is the first to examine the behaviour of the general population in Akatsi South District, Ghana, regarding hypertension and diabetes using the KAP questionnaire. Also, we reduced the likelihood of residual confounding in our models by adjusting for any possible bias. Finally, our sample size was good enough to draw meaningful conclusions. Our study, on the other hand, had limitations. First and foremost, weight and height are the only anthropometric parameters measured in this study, and using the BMI is not the best indicator of obesity, but analyzing the body composition will be better in this case. In addition, the participants measured their body weight, usually accompanied by intentional and non-intentional personal errors. Nevertheless, previous studies have reported that self-reported weight and height are acceptable for determining BMI (33). Finally, knowledge about possible symptoms of both HT and DM was not considered in the survey, so we limited attitude questions to three.

### 4.2. Study recommendations

Preventive behaviours may be encouraged when people have adequate knowledge about a disease to make them aware of its dangers. Hence, based on the findings of this study, we, therefore, recommend the district health directorate and healthcare professionals adopt periodic public health education and promotion via the mainstream media and community-to-community education to help bridge the knowledge gap.

## 5. Conclusion

Individuals can minimize early-stage disease mortality with adequate knowledge, attitudes, and practices. Significantly low levels of knowledge, attitude, and practice, which are challenges to eliminating determinants contributing to NCDs, were found in HT and DM in this district. Also, overweight and obese individuals should be a focus of health education as weight is a risk factor; meanwhile, they rarely or never check their blood pressure or sugar level. For people to be aware of the necessity of regular blood pressure and blood glucose screening as a preventive strategy and to avoid an increase in cardiovascular mortality, there is an urgent need to create awareness and educate the population accordingly.

## Data availability statement

The raw data supporting the conclusions of this article will be made available by the authors, without undue reservation.

## Ethics statement

The studies involving human participants were reviewed and approved by Ghana Health Service (GHS) Ethical Review Committee (ERC)-GHS-ERC: 17/02/2020. The patients/participants provided their written informed consent to participate in this study.

## Author contributions

DA and AD conceived and designed the study. DA, AW, and SK collected the data and performed the analysis of all data. DA and ZZ provided a review of the previous literature. DA, AD, AW, ZZ, SK, RL, KH, and JZ all participated in the discussion of the results. DA, AD, AW, and ZZ wrote all versions of the manuscript and figures. DA, ZZ, and JZ polished this manuscript, including grammatical checks. All authors contributed to manuscript revision, read, and approved the submitted version.

## Funding

This work was supported by the National Natural Science Foundation of China (grant 82173899).

## Conflict of interest

The authors declare that they conducted the research without any commercial or financial relationships that could be construed as a potential conflict of interest.

## Publisher’s note

All claims expressed in this article are solely those of the authors and do not necessarily represent those of their affiliated organizations, or those of the publisher, the editors and the reviewers. Any product that may be evaluated in this article, or claim that may be made by its manufacturer, is not guaranteed or endorsed by the publisher.

## References

[1] Gakidou E Afshin A Abajobir AA Abate KH Abbafati C. Global, regional, and national comparative risk assessment of 84 behavioural, environmental and occupational, and metabolic risks or clusters of risks, 1990-2016: a systematic analysis for the global burden of disease study 2016. Lancet. (2017) 390:1345–422. doi: 10.1016/S0140-6736(17)32366-8, PMID: 28919119 PMC5614451

[2] Noncommunicable diseases (n.d.). Available at: https://www.who.int/news-room/fact-sheets/detail/noncommunicable-diseases (Accessed April 21, 2022).

[3] Mathers CD Loncar D. Projections of global mortality and burden of disease from 2002 to 2030. PLoS Med. (2006) 3:e442. doi: 10.1371/journal.pmed.0030442 17132052 PMC1664601

[4] Yach D Hawkes C Gould CL Hofman KJ. The global burden of chronic diseases: overcoming impediments to prevention and control. JAMA. (2004) 291:2616–22. doi: 10.1001/jama.291.21.2616, PMID: 15173153

[5] Imran M Hashmi A. Knowledge, attitudes and practices of patients regarding knowledge, attitudes and practices of patients (2019) 32:166–72.

[6] Budreviciute A Damiati S Sabir DK Onder K Schuller-Goetzburg P Plakys G. Management and prevention strategies for non-communicable diseases (NCDs) and their risk factors. Front Public Heal. (2020) 8:788. doi: 10.3389/fpubh.2020.574111 PMC7726193 33324597

[7] IDF (2021). IDF Diabetes Atlas | Tenth Edition, Int. Diabetes Fed. Available at: https://diabetesatlas.org/ (Accessed April 21, 2022).

[8] Anyanti J Akuiyibo SM Fajemisin O Idogho O Amoo B. Assessment of the level of knowledge, awareness and management of hypertension and diabetes among adults in Imo and Kaduna states, Nigeria: a cross-sectional study. BMJ Open. (2021) 11:e043951. doi: 10.1136/bmjopen-2020-043951, PMID: 34006029 PMC7942260

[9] Bosu WK Bosu DK. Prevalence, awareness and control of hypertension in Ghana: a systematic review and meta-analysis. PLoS One. (2021) 16:e0248137. doi: 10.1371/journal.pone.0248137 33667277 PMC7935309

[10] Gatimu SM Milimo BW Sebastian MS. Prevalence and determinants of diabetes among older adults in Ghana. BMC Public Health. (2016) 16:1–12. doi: 10.1186/s12889-016-3845-8 27871259 PMC5117548

[11] Sory E. Ghana Health Service Annual Report (2009). Ghana: Ministry of Health, 61 p.

[12] Agyei-Mensah S De-Graft Aikins A. Epidemiological transition and the double burden of disease in Accra, Ghana. J Urban Health. (2010) 87:879–97. doi: 10.1007/s11524-010-9492-y 20803094 PMC2937133

[13] Qureshi NN Hatcher J Chaturvedi N Jafar TH. Effect of general practitioner education on adherence to antihypertensive drugs: cluster randomised controlled trial. BMJ Br Med J. (2007) 335:1030. doi: 10.1136/bmj.39360.617986.AE 17991935 PMC2078673

[14] WHO Global Report, Global report on diabetes, ISBN. 978 (2016), 11. (Accessed May 17, 2022).

[15] Mills KT Bundy JD Kelly TN Reed JE Kearney PM Reynolds K. Global disparities of hypertension prevalence and control: a systematic analysis of population-based studies from 90 countries. Circulation. (2016) 134:441–50. doi: 10.1161/CIRCULATIONAHA.115.018912 27502908 PMC4979614

[16] Tarkang E Atinyi R Takramah W Axame WK Owusu R Parbey PA. Prevalence and awareness of hypertension among urban and rural adults in the Keta municipality, Ghana. J Med Res. (2017) 3:155–63. doi: 10.31254/jmr.2017.3313

[17] Asante DO Walker AN Seidu TA Kpogo SA Zou J. Hypertension and diabetes in Akatsi South District, Ghana: modeling and forecasting. Biomed Res Int. (2022) 2022:1–11. doi: 10.1155/2022/9690964, PMID: 35187174 PMC8850043

[18] D.P.C.-O.U.-A.S.D. Assembly, Ministry of Local Government and Rural Development Akatsi South District Assembly, (2017) 2014–2017. ASD, Pennsylvania.

[19] Ghana Statistical Service. Akatsi South District, 2010 Popul. Akatsi Sou: Census (2014).

[20] Kadam P Bhalerao S. Sample size calculation. Int J Ayurveda Res. (2010) 1:55. doi: 10.4103/0974-7788.59946 20532100 PMC2876926

[21] Pourhoseingholi MA Vahedi M Rahimzadeh M. Sample size calculation in medical studies. Gastroenterol Hepatol Bed Bench. (2013) 6:14–7. 24834239 PMC4017493

[22] Frantz J. A knowledge assessment questionnaire relating to risk factors for chronic disease of lifestyle for high school learners: validity and reliability. J Community Heal Sci. (2008) 3:30–7.

[23] Weir C.B. Jan A., BMI Classification Percentile and Cut Off Points, StatPearls. (2021). Available at: https://www.ncbi.nlm.nih.gov/books/NBK541070/ (Accessed April 12, 2022). 31082114

[24] NHLBI Obesity Education Initiative, The Practical Guide Identification, Evaluation, and Treatment of Overweight and Obesity in Adults NHLBI, (2000) 9–11. Bethesda

[25] De-Graft Aikins M Kushitor K Koram S Gyamfi GO. Chronic non-communicable diseases and the challenge of universal health coverage: Insights from community-based cardiovascular disease research in urban poor communities in Accra, Ghana. BMC Public Health. (2014) 14:S3. doi: 10.1186/1471-2458-14-S2-S3 PMC4120153 25082497

[26] Kayima J Wanyenze RK Katamba A Leontsini E Nuwaha F. Hypertension awareness, treatment and control in Africa: a systematic review. BMC Cardiovasc Disord. (2013) 13:1–11. doi: 10.1186/1471-2261-13-1 23915151 PMC3750220

[27] WHO. 2008-2013 Action Plan for the Global Strategy for the Prevention and Control of Noncommunicable Diseases. Geneva: WHO (2009).

[28] Dussa K Parimalakrishnan S Sahay R. Assessment of diabetes knowledge using diabetes knowledge questionnaire among people with type 2 diabetes mellitus. Asian J pharm Clin Res. (2015) 8:254–6.

[29] Arugu GM Maduka O. Risk factors for diabetes mellitus among adult residents of a rural district in southern Nigeria: implications for prevention and control. Niger J Clin Pract. (2017) 20:1544–9. doi: 10.4103/njcp.njcp_154_17, PMID: 29378984

[30] Chien SY Chuang MC Chen IP. Why people do not attend health screenings: factors that influence willingness to participate in health screenings for chronic diseases. Int J Environ Res Public Health. (2020) 17. doi: 10.3390/ijerph17103495, PMID: 32429532 PMC7277138

[31] Oguoma VM Nwose EU Skinner TC Digban KA Onyia IC Richards RS. Prevalence of cardiovascular disease risk factors among a Nigerian adult population: relationship with income level and accessibility to CVD risks screening. BMC Public Health. (2015) 15:1–16. doi: 10.1186/s12889-015-1709-2 25925238 PMC4415344

[32] Kassie AM Abate BB Kassaw MW Aragie TG Geleta BA Shiferaw WS. Impact of knowledge and attitude on the utilization rate of cervical cancer screening tests among Ethiopian women: a systematic review and meta-analysis. PLoS One. (2020) 15:e0239927. doi: 10.1371/journal.pone.0239927, PMID: 33290426 PMC7723289

[33] Davies L Wellard-Cole A Rangan M. Allman-Farinelli, validity of self-reported weight and height for BMI classification: a cross-sectional study among young adults. Nutrition. (2020) 71:110622. doi: 10.1016/j.nut.2019.110622 31837644

